# Epstein-Barr virus infection: the micro and macro worlds

**DOI:** 10.1186/s12985-023-02187-9

**Published:** 2023-10-02

**Authors:** Wei Huang, Lang Bai, Hong Tang

**Affiliations:** 1 Center of Infectious Diseases, West China Hospital of Sichuan University, Chengdu, 610041 China; 2 Division of Infectious Diseases, State Key Laboratory of Biotherapy and Center of Infectious Diseases, West China Hospital, Sichuan University, Chengdu, 610041 China

**Keywords:** Immune response, Immune escape, EBV-associated diseases, Epstein‒Barr virus

## Abstract

Epstein‒Barr virus (EBV) is a DNA virus that belongs to the human B lymphotropic herpesvirus family and is highly prevalent in the human population. Once infected, a host can experience latent infection because EBV evades the immune system, leading to hosts harboring the virus for their lifetime. EBV is associated with many diseases and causes significant challenges to human health. This review first offers a description of the natural history of EBV infection, clarifies the interaction between EBV and the immune system, and finally focuses on several major types of diseases caused by EBV infection.

## Introduction

Epstein‒Barr virus (EBV), as a gammaherpesvirus, is a widely distributed oncogenic virus that was first identified in a biopsy of a patient with Burkitt lymphoma [1]. As a human lymphotropic herpesvirus, it is also the first virus identified by the World Health Organization (WHO) to cause cancer. Epidemiological studies have shown that the infection rate of EBV in the population exceeds 95% [2]. EBV is mainly spread through saliva. Exposure to bodily fluids, breast milk, and EBV-positive organ transplantation contributes to the spread of the virus [3]. There are two EBV subtypes that can infect humans: EBV-1 and EBV-2; these subtypes differ in their EBV nuclear antigen-2 (EBNA-2) and EBNA-3 gene sequences (EBNA-3a, EBNA-3b and EBNA-3c) [4, 5]. EBV-1 is widely distributed and efficiently transforms B lymphocytes into immortalized LCLs (lymphoblastoid cell lines) in vitro [6], whereas EBV-2 is predominantly found in Africa and is more likely to infect cultured T cells than B cells [7, 8].

EBV infection is divided into three main phases: primary infection and lytic replication, latency and lytic reactivation [9]. Most primary EBV infections occur in infants and children. In North China, the seroprevalence of anti-EBV antibodies in children can reach more than 80% [10]. Infections in childhood are usually asymptomatic or present as an upper respiratory infection, but later, these infections often lead to infectious mononucleosis (IM) [11]. After primary infection, the virus remains dormant, with memory B cells serving as the main reservoir of their persistence [12]. Most people are in the latent phase of EBV infection and show no health-threatening clinical manifestations. However, when human immunity is weakened, many EBVs can be activated and enter the lytic reactivation phase, causing specific diseases. EBV infection mainly causes four types of diseases: IM, chronic active EBV infection (CAEBV), EBV-associated autoimmune disease and EBV-associated tumorigenesis. EBV-associated diseases seriously threaten human health, and research on how EBV persists in the host and how to effectively clear EBV is currently intensive. This review first presents the natural history of EBV infection, then clarifies the interaction between EBV and immunity, and finally focuses on several major types of diseases caused by EBV.

## Natural history of EBV infection

**Fig. 1:**
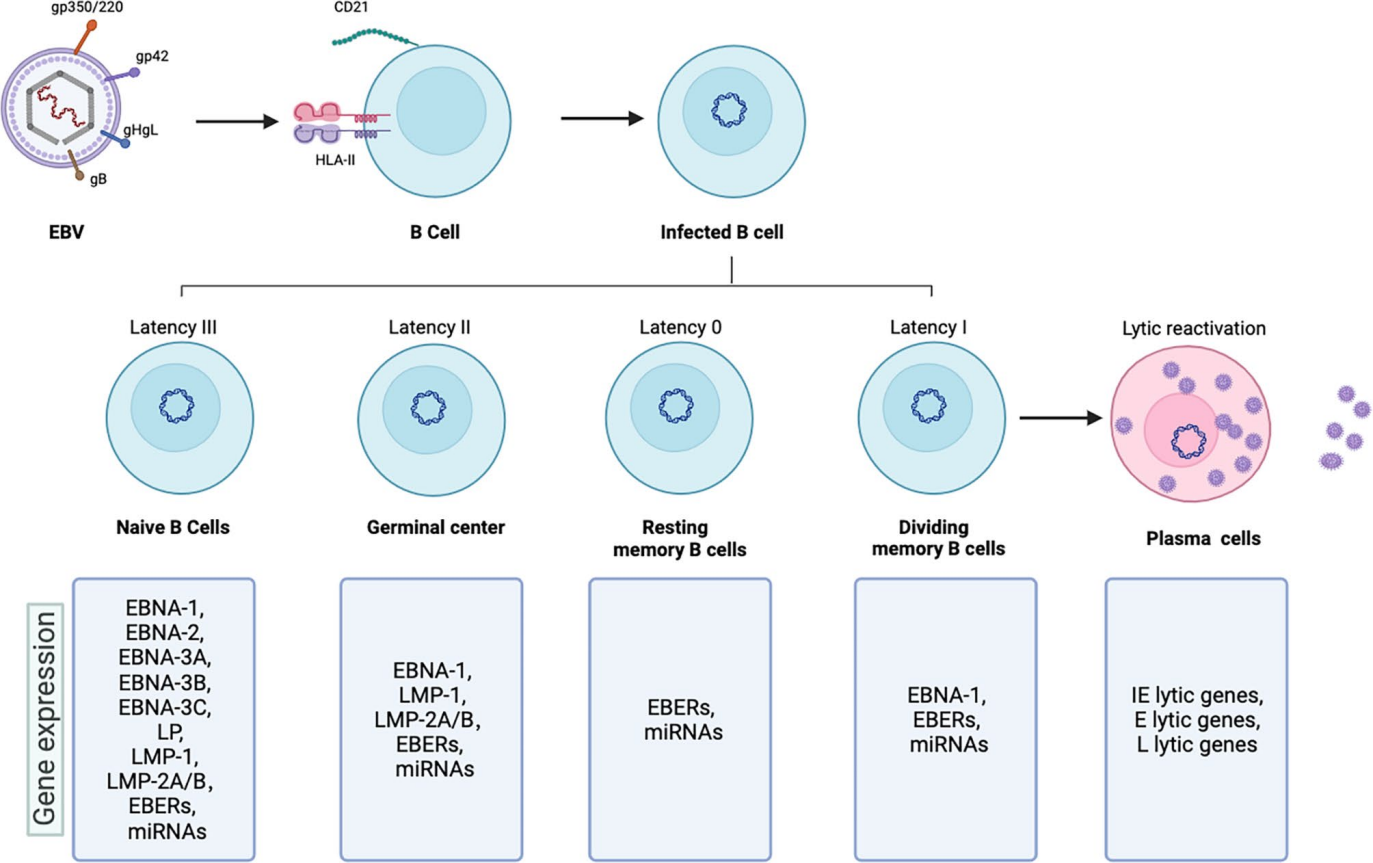
Major interaction modes of EBV membrane glycoproteins with B cells and the natural history of EBV infection

EBV particles are spherical and consist of envelope, tegument and nucleocapsid in the outside-to-inside direction. Glycoproteins are attached to the envelope, and eight of them participate in EBV invasion, which is essential for infecting a host [13]; the tegument contains unevenly distributed tegument proteins, and it is a unique structure in herpesviruses; the nucleocapsid is an icosahedron formed from multiple capsid proteins that enclose a 172-kpb double-stranded DNA genome [14]. EBV carries more than 100 genes encoding approximately 85 proteins and approximately 50 noncoding RNAs [15]. (Fig. 1)

### The primary infection and lytic replication phases

EBV is mainly transmitted through saliva, and the virus initially infects B cells and epithelial cells in the oropharynx. Different glycoproteins of EBV are involved in infecting B cells or epithelial cells. Glycoprotein gp350/220 binds to complement receptor type 2 (CR2/CD21) on B cells to advance viral attachment. In addition to the protein binding CD21, another gp350/220 receptor, complement receptor type 1 (CR1/CD35), can also bind B cells [3, 16]. Following attachment, EBV glycoprotein gp42 can interact with HLA class II (HLA-II) on B cells, bringing the virus close to the cell and then triggering the core fusion machinery consisting of gHgL and gB to interact with endosomal membranes. Because epithelial cells lack CD21 and HLA-II, viral gHgL and gB appear to be involved in EBV entry and fusion with epithelial cells [17]. After fusion, the tegument with the nucleocapsid is released into the cytoplasm [18]. EBV fusion with B cells and epithelial cells involve three envelope glycoproteins that play major roles, including gHgL and gB [19]. Since these proteins are conserved throughout the herpesvirus family, they are also referred to as the core fusion machinery [13]. Initially, investigators believed that EBV infects epithelial cells through direct membrane fusion [20]. However, a recent study found that EBV also enters epithelial cells through lipid raft-dependent endocytosis and micropinocytosis [21].

After the tegument proteins of the virus are dissolved, the EBV genome is injected into the nucleus, and viral replication is mediated by DNA polymerase; the whole process occurs during the lytic phase of the viral life cycle. The EBV genome encodes over 80 gene products that facilitate viral replication and the synthesis of viral structural components during the lytic phase. [22]. The temporal sequence of viral gene expression can be classified into three steps: immediate early (IE), early (E) and late (L) [23]. IE lytic genes, such as *BZLF1* and *BRLF1*, encode transactivators of the viral lytic program [24]. E lytic genes, such as *BNLF2a*, are related mainly to virus replication and metabolism. L lytic genes, such as *BCRF1*, may be related to immune escape [25]. Simultaneously, EBV activates growth transformation programs to drive infected B-cell proliferation and differentiation of infected B cells into memory B cells in a germinal center reaction. In addition, antigen-presenting cells present antigenic substances to T cells. Infected B cells are then attacked by cytotoxic T lymphocytes (CTLs), which release viral particles into the peripheral circulatory system and control the number of infected B cells [26–28]. Infected memory B cells are also released into the peripheral circulation where can remain in the latent or lytic replication phase. EBV infects and replicates in epithelial cells and B cells locally infiltrating the oral cavity, resulting in the shedding of large amounts of virus in the oropharynx and the entry of infected B cells into the bloodstream, circulating between the oral cavity and peripheral vascular system [29].

### Latent infection phase

EBV can maintain in the latent infection phase in the human host, which is an important reason why the virus cannot be eradicated and can maintain lifelong persistence. After primary infection, the linear EBV DNA becomes circular DNA in the host cell nucleus in the form of circular episomes [30]. The circular episomes attach to host chromatin through the action of EBNA-1, replicates in conjunction with the host cell cycle, and is transmitted to daughter cells [31]. During the latent infection phase, EBV expresses only a limited subset of viral genes and noncoding RNAs, including six nuclear antigens, EBNA-1, EBNA-2, EBNA-3 A, EBNA-3B, EBNA-3 C, and LP; three latent membrane proteins (LMPs), LMP-1 and LMP-2 A/B; and two types of non-coding RNA that are not translated into proteins, EBV-encoded RNAs (EBERs) and microRNAs (miRNAs) [4, 32]. EBV is specific and unique because of its ability to establish different latent gene expression patterns (the Latency Type 0, I, II and III) depending on the infected cell type and state (resting or proliferating) [27]. The latent EBV genome propagates in dividing memory B cells throughout the Latency I period [33], induces B-cell differentiation through the Latency II period [34], indues the growth and transformation of naive B cells through Latency III genes [35], and stops all viral gene expression in the Latency 0 period in the memory B-cell pool. Different latent gene expression programs express different viral gene products.

### Lytic reactivation phase

Infected memory B cells in the latent infection phase are occasionally reactivated and differentiated into plasma cells, which can induce EBV reactivation after cytolysis, thereby entering the lytic reactivation phase from the latent infection phase [36]. *BZLF1* and *BRLF1*, IE lytic genes, encode transcription factors Z (also named ZTA/BZLF1) and R (also named RTA/BRLF1), which play important roles in EBV reactivation [37, 38]. The expression of BZLF1 and BRLF1 in the latent phase is inhibited by a variety of cellular transcriptional repressors. During the lytic replication phase, BZLF1 and/or BRLF1 proteins bind to the origin site of EBV DNA replication (named *oriLyt*) in the genome as initial binding proteins [39], activate their own and each other’s promoters (Zp and Rp), and ultimately cooperate to activate the promoter of the E lytic gene [40]. When the replication of the EBV genome is complete, the new viral genome, which is unmethylated and has no chromatin, is linearized by the viral terminase complex and subsequently packaged into preformed capsid particles to form nucleocapsids. A nucleocapsid exits the nucleus and enters the cytoplasm where it is coated with viral proteins to form the tegument. The virus particle finally acquires an envelope under the action of the Golgi apparatus. Through exocytosis, cellular vesicles fuse with the plasma membrane and release mature and infectious virions [3, 41].

## The interaction of the immune system and EBV

The interaction between immune factors and EBV is complex. On the one hand, the immune system plays a critical role in controlling EBV infection. When a person is infected with EBV, the immune system produces specific antibodies that target the virus and help to clear it from the body. However, EBV can evade the immune system in various ways, allowing it to establish persistent infection in the body. EBV can interfere with immune system function, making it harder for the body to fight the infection.

### Immune responses to EBV

#### Innate immunity

**Fig. 2:**
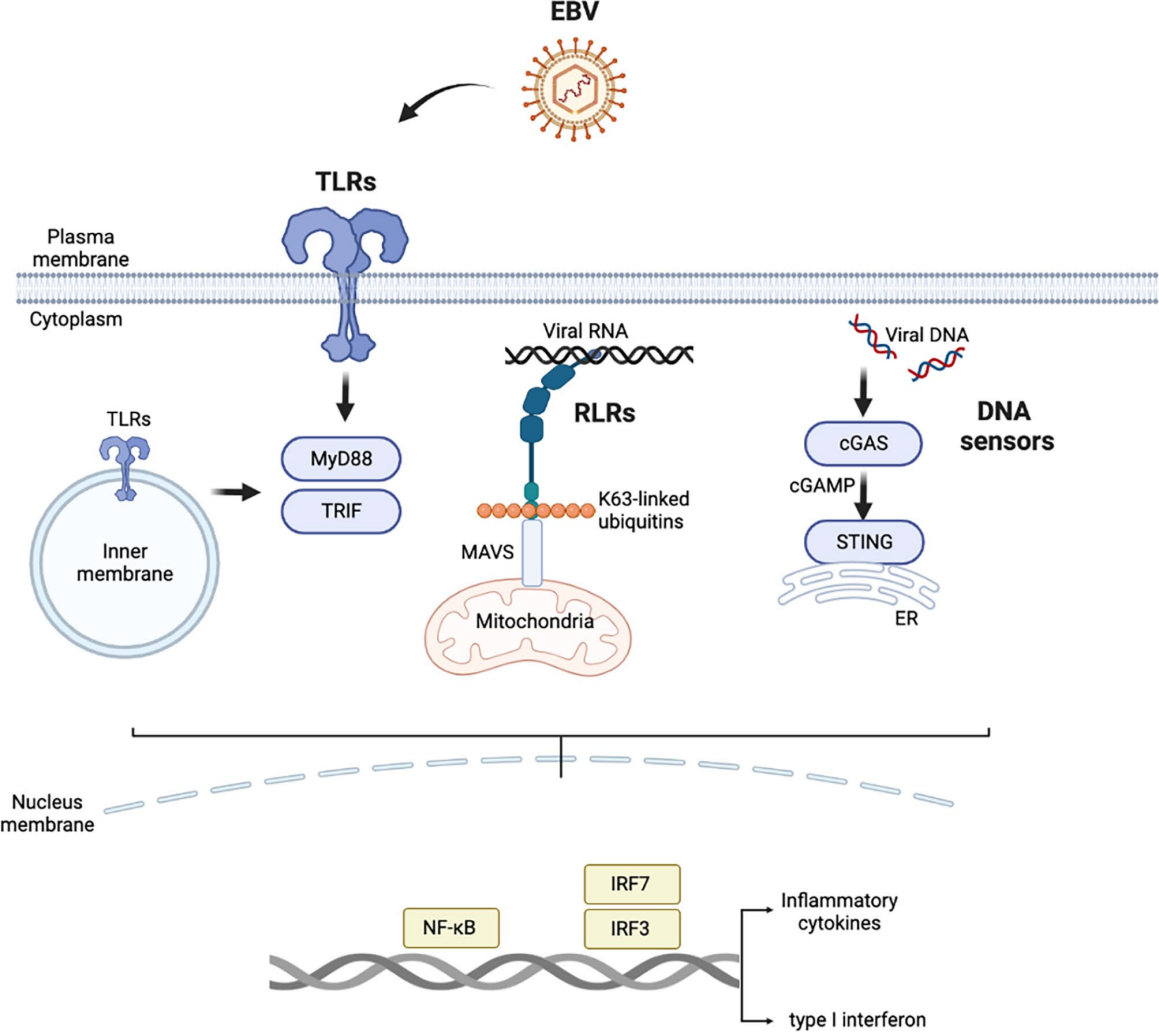
Major pattern recognition receptors in the innate immune response to EBV. Abbreviations: EBV, Epstein‒Barr virus; TLRs, Toll-like receptors; MyD88, myeloid differentiation primary response 88; TRIF, TIR-domain-containing adapter protein-inducing interferon-β; RLRs: retinoic acid-inducible gene-I-like receptors; MAVS, mitochondrial antiviral signaling; cGAS: cyclic GMP-AMP synthase; cGAMP, cyclic GMP-AMP; STING, stimulator of interferon genes; ER, endoplasmic reticulum; IRF, interferon regulatory factor

As the body’s first line of defense against EBV invasion, innate immunity not only resists nonspecific infection but also initiates the process of adaptive immunity in which it also participates [26]. Innate immune responses are induced after recognition of biomacromolecules with pathogen-associated molecular patterns (PAMPs) or damage-associated molecular patterns (DAMPs) by pattern recognition receptors (PRRs). PAMPs include a number of biological macromolecules, such as DNA, RNA, and lipids, carried by invading EBV. PRRs that specifically recognize EBV PAMPs mainly include Toll-like receptors (TLRs), retinoic acid-inducible gene-I (RIG-I)-like receptors (RLRs), and a series of intracellular DNA sensors, including cyclic GMP-AMP (cGAMP) synthase (cGAS) and interferon gamma-inducible protein 16 (IFI16). After PRRs are activated, a series of signaling cascades are triggered to produce various cytokines, chemokines and/or type I interferon (IFN-I), which inhibit protein translation and growth of infected cells, promote apoptosis, limit virus spread, and activate adaptive immunity [42]. (Fig. 2)

TLRs are distributed mainly on the plasma membrane and membranes inside cells, and all TLR signaling is transduced mainly through one of two adapter proteins, namely, myeloid differentiation primary response 88 (MyD88) and TIR-domain-containing adapter protein-inducing interferon-β (TRIF), although differences in signaling are profound [43, 44]. EBV infection mainly activates TLR2, TLR3, TLR7 and TLR9 [45]. Different TLRs respond to different PAMPs, but they all ultimately activate the NF-κB signaling pathway to produce proinflammatory cytokines [such as interleukin (IL)-1, IL-6, IL-8, and tumor necrosis factor-α (TNF-α)] and induce interferon regulatory factors (IRF) 3 and IRF7 to produce IFN-I. This mechanism helps to control the spread of the virus and limit the development of EBV-associated diseases.

RLRs can recognize foreign RNA in the cytoplasm, and a study showed that RLRs recognize EBERs through an RNA polymerase III-dependent pathway and then activate the inflammatory response triggered by the NF-κB and IRF3 signaling pathways [46]. After knocking down RIG-1 in gastric cancer cell lines, the production of inflammatory factors after EBV reactivation was found to be significantly reduced, further proving that RLRs play roles in fighting EBV infection [47].

At present, few studies on how DNA sensors identify EBV have been reported. As a DNA sensor, cGAS recognizes and binds double-stranded DNA (dsDNA) in a length-dependent manner. After binding dsDNA, cGAS catalyzes adenosine triphosphate (ATP) and guanosine triphosphate (GTP) to produce cGAMP, and then, cGAMP binds and activates stimulator of interferon genes (STING) to secrete IFN-I [48]. Although EBV-positive B cells express cGAS and stimulator of interferon genes (STING), no evidence to suggest that downstream IFN production is induced has been reported [49]. When the transcription of another DNA sensor, IFI6, is knocked down, EBV replication is increased in B cells [50].

Some innate immune cells recognize EBV. EBV infection can promote the proliferation of natural killer (NK) cells, and this expanded population of NK cells can recognize and lyse infected cells [51]. In addition, invariant NKT (iNKT) cells can restrict the ex vivo transformation of B lymphocytes induced by EBV [52]. In addition, as antigen-presenting cells (APCs), such as dendritic cells (DCs), not only recognize pathogens via innate immunity programs but also play crucial roles in adaptive immunity by activating immune cells. DCs can be classified into plasmacytic dendritic cells (pDCs) and classical myeloid dendritic cells (cDCs) [53]. pDCs express TLR7 and TLR9, which recognize EBV nucleic acids and produce IFN-I [54, 55]; cDCs recognize EBV through TLR3 and can process and present antigens to T cells [56].

#### Adaptive immunity

Adaptive immunity is the arm of the immune system that is specifically tailored to recognize and respond to foreign antigens, including viral antigens. The adaptive immune response to EBV infection involves both B cells and T cells.

B cells participate in adaptive immune responses and produce specific antibodies. Anti-viral capsid antigen (VCA) immunoglobulin M (IgM) and IgA antibodies are produced early in the infection period and persist for several weeks to months before disappearing, whereas anti-VCA IgG antibodies typically peak 2–4 months after infection, and then, although the number declines, persist in the body. Infected B cells also produce antibodies against gp350, gp42 and gHgL, inhibiting EBV binding B cells and viral fusion, limiting viral spread, and preventing recurrent infection [57–59].

The generation of EBV-specific CD8 + and CD4 + T cells is significantly elevated in the population of EBV-infected individuals. CD8 + cytotoxic T cells target and attack EBV-infected cells by recognizing viral protein peptides presented by major histocompatibility complex Class I molecules (MHC-I) on infected cells and play antiviral roles [60]. Mediated via these cytotoxic T cells, a single lytic antigen-specific CD8 + T-cell response can involve up to 2% of the CD8 + T-cell population, and latent antigen-specific responses involve only 1% of the CD8 + T-cell population [61]. Studies have revealed that CD8 + T cells show specific affinity for epitopes in IE lytic gene products, with lower specific affinity for E lytic gene products and very little for epitopes in L lytic gene products [62]. Latent responses are mainly directed against epitopes in the EBNA3 protein family, to a lesser extent against LMP2, EBNA1 and EBNA2 protein epitopes and very rarely against EBNA-LP pr LMP1 [61]. The EBNA3 protein family specifically causes the expansion of CD8 + cytotoxic T cells, thereby inhibiting the excessive growth, proliferation, and tumor formation of transformed B cells [63]. EBV-positive posttransplant B lymphoproliferative disease (PTLD) is more likely to develop in patients with suppressed T-cell function, such as those with myelosuppression or who undergo organ transplantation [64]. However, PTLD can be successfully treated the by adoptive transfer of EBV-specific T-cell preparations [60]. B cells infected with EBV express high levels of MHC-II molecules, which activate CD4 + T cells. CD4 + T cells not only assist B cells in producing antibodies and neutralizing antigens but also induce and maintain the cytotoxic activity of CD8 + T cells. In contrast to CD8 + T cells, CD4 + T cells are less likely to respond to individual epitopes. Thus, latent antigen-specific CD4 + T-cell responses are more robust than their lytic antigen-specific responses. Finally, the antigen-specific CD4 + T-cell responses to IE, E, and L lytic gene products are evenly activated [61]. Accumulating evidence shows that CD4 + T cells can also act as direct effector cells to recognize and kill newly EBV-infected B cells or established EBV-transformed LCLs [65, 66].

### Evasion of immunity by EBV

#### Innate immunity

To achieve long-term survival in the host and establish persistent infection, EBV has also evolved many strategies to evade host immune surveillance. First, EBV can downregulate the activation of several PRRs. Second, it can also directly target downstream factors. Finally, it can affect the function of some immune cells. (Table 1)

**Table 1:** EBV immune evasion proteins and microRNAs.

EBV gene	Function /Phase /Location	Evasion mechanism	Ref.
LMP1	Latency II	Reducing TLR9 promoter activity; decreasing TLR9 mRNA and protein expression levels	[68]
		Reducing the phosphorylation of Tyk2 and STAT2; inhibiting IFN pathway activation	[78]
BGLF5	Lytic immune modulator	Depleting TLR9 mRNA and reducing its protein expression level	[69]
		Inducing host mRNA degradation; blocking the synthesis of MHC-I	[90]
BPLF1	Tegument	Removing ubiquitin tags from TRAF6; negatively regulating TLR signaling	[45]
		Mediating DUB-dependent deubiquitination of TBK1 and STING; inhibiting RIG-I-MAVS and cGAS-STING signaling	[72]
BHRF1	Lytic immune modulator	Inducing mitochondrial fission; causing MAVS protein degradation; blocking RLRs signaling	[70]
miR-BART6-3p	EBV-encoded microRNA	Targeting the 3’UTR of RIG-I mRNA; inhibiting the expression of IFN-β	[71]
BZLF1 (ZTA)	Lytic replication	Binding directly to IRF7; inhibiting IRF7 activation	[75]
		Upregulating SOCS3 expression; indirectly inhibiting IFN-α production	[76]
		Binding to CIITA; inhibiting MHC-II transcription	[93]
BRLF1 (RTA)	Lytic replication	Reducing the mRNA levels of IRF 3 and IRF7 and the activation of the IFN-β promoter; inhibiting the expression of IFN-β	[77]
LMP2	Latency II	Reducing the phosphorylation of Tyk2, STAT1 and JAK; inhibiting ISG transcription and the IFN production	[79]
LF2	Lytic immune modulator	Binding to IRF7 to block its dimerization; inhibiting IFN-α production	[80]
BGLF4	Late gene expression	Reducing the activity of IFN-β promoter; inhibiting IRF3 signaling	[81]
		Interfering with the interaction between NF-κB and UXT; inhibiting the activity of NF-κB	[82]
		Phosphorylating SAMHD1; decreasing the activity of dNTPase	[83]
miR-BART18-5p	EBV-encoded microRNA	Targeting MAP3K2; blocking viral replication	[86]
miR-BART4-5p	EBV-encoded microRNA	Downregulating proapoptotic protein BID activity; inhibiting target cells apoptosis	[87]
EBNA1	Latency I	Inhibiting ULBP1 and ULBP5; escaping NK cell recognition	[88]
BNLF2a	Lytic immune modulator	Inhibiting TAP function; preventing loading of antigenic peptides onto MHC-I	[91]
BILF1	Lytic immune modulator	Triggering endocytosis of MHC-I molecules and degradation	[92]
BZLF2	Entry glycoprotein	Binding to MHC-II complex; blocking the antigen recognition of CD4 + T cells	[94]

Abbreviation: EBV, Epstein‒Barr virus; LMP1: latent membrane protein 1; TLR: Toll-like receptor; Tyk: tyrosine kinase; STAT, transducer and activator of transcription; IFN, interferon; MHC-I/II: major histocompatibility complex class I/II molecules; TLR, Toll-like receptor; DUB, deubiquitinase; RIG-I: retinoic acid-inducible gene-I; RLRs: RIG-I -like receptors; MAVS, mitochondrial antiviral signaling; cGAS: cyclic GMP-AMP synthase; cGAMP, cyclic GMP-AMP; STING, stimulator of interferon genes; ER, endoplasmic reticulum; IRF, interferon regulatory factor; CIITA, class II transactivator; ISG, interferon-stimulated gene; IFN, interferon; SAMHD1: sterile alpha motif and HD domain 1; dNTPase: deoxynucleotide triphosphate hydrolase; EBNA: EBV nuclear antigen; ULBP, UL16-binding proteins; NK, natural killer; TAP, transporter associated with antigen processing.

EBV can reduce TLR expression and/or signal transduction to escape cellular immune responses to TLR activation [67]. Fathallah et al. found that the EBV oncoprotein LMP1 is a strong inhibitor of TLR9 transcription, and its overexpression regulates NF-κB pathway activation, thereby reducing TLR9 promoter activity and its mRNA and protein expression levels [68]. Moreover, the EBV exonuclease BGLF5, a protein kinase constituent in all herpesviruses, evades host immune signaling by depleting TLR9 mRNA levels and thereby reducing TLR9 protein expression [69]. Due to its deubiquitinase (DUB) activity, the EBV large tegument protein BPLF1 can negatively regulate TLR signaling by removing ubiquitin tags from proteins in the TLR signaling cascade [45].

Oligomeric RIG-I or MDA5 (an RLR family protein), with action mediated by K63-linked ubiquitin chains, interacts with the N-terminal caspase recruitment domain in mitochondrial antiviral signaling (MAVS) located on the mitochondrial membrane and induces RLR-mediated signal transduction. As an anti-apoptotic protein, EBV BHRF1 can induce mitochondrial fission, cause mitochondrial and MAVS protein degradation, block RLR-mediated signal transduction, and weaken antiviral effects [70]. As an EBV-encoded microRNA, miR-BART6-3p can target the 3’UTR of RIG-I mRNA, thereby inhibiting the expression of IFN-β [71]. BPLF1 also represses the expression of the RIG-I-MAVS and cGAS-STING pathways via the DUB-dependent deubiquitination of TBK1 and STING [72].

Moreover, EBV can target DNA sensors. EBV induces the body to produce the E3 ubiquitin ligase tripartite motif-containing protein 29 (TRIM29), which can degrade STING, and the downregulation of STING may inhibit the production of IFN-I and the innate immune response [73].

In addition to affecting the activation of several PRRs, EBV can also directly target downstream factors to escape the immune system [74]. Studies have revealed that BZLF1 can directly or indirectly downregulate the expression of IRF7 to inhibit the production of IFN-α [75, 76]. BRLF1 inhibits the production of IFN-β by reducing the mRNA levels of IRF 3 and IRF7 and reducing the activation of the IFN-β promoter [77]. LMP-1 inhibits the IFN pathway by reducing the phosphorylation of tyrosine kinase 2 (Tyk2) and signal transducer and activator of transcription 2 (STAT2) [78]. LMP-2 A/B inhibited interferon-stimulated gene (ISG) transcription and IFN production by reducing the phosphorylation of Tyk2, STAT1 and JAK [79]. As an EBV tegument protein, LF2 inhibits IFN-α production by binding to IRF7 to block its dimerization [80]. As a viral protein kinase, BGLF4 not only inhibits IRF3 and NF-κB signaling [81, 82] but also phosphorylates sterile alpha motif and HD domain 1 (SAMHD1), resulting in a decrease in its deoxynucleotide triphosphate hydrolase (dNTPase) activity, allowing EBV to evade host immunity [83]. Studies have found that cellular dsRNA-dependent protein kinase (PKR) plays a key role in antiviral innate immunity, and EBERs can bind to PKR and inhibit its activation, thereby preventing PKR-mediated apoptosis [84]. EBV encodes at least 40 kinds of miRNAs, which are located in the *BHRF1* gene and BART transcription sequence in the form of gene clusters [85]. In addition to the abovementioned miR-BART6-3p, other miRNAs encoded by EBV, mainly miR-BART18-5p and miR-BART4-5p, evade host immunity by maintaining their presence in the latent state and inhibiting the apoptosis of infected cells [86, 87].

EBNA1 enables newly infected B cells to escape NK cell recognition by inhibiting NK cell receptor ligands UL16-binding protein 1 (ULBP1) and ULBP5 [88]. EBV evades the immune response by upregulating the expression of T-cell inhibitory factors so that infected pDCs cannot induce a T-cell response [89].

#### Adaptive immunity

Notably, EBV has evolved many strategies to evade adaptive immunity. On the one hand, EBV can interfere with MHC-I antigen presentation. The proteasome produces antigenic peptides, transports them to the endoplasmic reticulum through the transporter-associated with antigen processing (TAP) complex, binds to MHC-I, and then transports the peptides to the cell surface, where they are recognized by CD8 + T cells. BGLF5 abrogates MHC-I gene expression through a host-mediated shutdown program [90]. BNLF2a abrogates the TAP-mediated import of antigenic peptides [91]. BILF1 triggers endocytosis of MHC-I molecules and their degradation in lysosomes [92]. On the other hand, EBV can interfere with MHC-I antigen presentation. Class II transactivator (CIITA) can promote the expression of MHC-II, and BZLF1 can bind to CIITA, which inhibits MHC-II transcription [93]. As a lytic phase protein, BZLF2 can block antigen recognition by CD4 + T cells by binding to the MHC-II complex on the surface of B cells [94]. It has been reported that EBV can also increase the number of specific regulatory T cells (Tregs), and the action of these EBV-specific Tregs may be related to the escape of tumor cells [95, 96].

## EBV-associated diseases

The diseases caused by EBV cover a wide range condition, from mild asymptomatic infection to tumorigenesis. They can be mainly divided into the categories discussed in this section.

### Primary EBV infection-associated diseases

IM is one of the most common manifestations of EBV infection. IM occurs in approximately 35-50% of people who are first infected with EBV in adolescence [97]. The main symptoms of IM include sore throat, fever, and enlarged lymph nodes, which may be accompanied by atypical lymphocytosis. These symptoms are mainly caused by the massive proliferation of CD8 + T cells activated against latent and lytic viral antigens, especially the action of the EBNA3 protein family and the IE lytic gene products, accompanied by the release of many inflammatory factors [98, 99]. EBV-specific antibody tests and heterophilic antibody tests can be used to diagnose acute EBV infection. EBV initially infects and induces the proliferation of B cells, and the disease process usually resolves as CD8 + T-cell responses are activated to eliminate Latency Type III-infected B cells [99]. Acyclovir can only inhibit the EBV lytic replication phase not the latent infection phase, so it cannot reduce the severity, shorten the course, or decrease the incidence of complications [100]. Therefore, symptomatic treatment is generally adopted for patients with IM, and most patients recover spontaneously, but in a few cases, the diseases progress with serious complications such as CAEBV and HLH.

### Persistent EBV infection-associated diseases

CAEBV is a progressive disease with a duration of ≥ 3 months and markedly elevated EBV DNA levels in the absence of immunodeficiency [101]. After infection, the main clinical manifestations are persistent or recurrent IM-like symptoms and progressive chronic damage to multiple organs, such as liver function damage, multiple lymphadenopathies, hepatosplenomegaly, HLH, retinitis, interstitial pneumonia, vaccinia-like vesicular disease, and mosquito bite allergies [102]. These outcomes are mainly caused by organ infiltration by EBV-infected lymphocytes. A prospective study revealed that EBV infected 3/5 of T cells and 2/5 of NK cells, and the predominant infiltrating immune cells were CD4 + T cells [102]. The major EBV pattern in T/NK cells followed Latency Type II infection and showed increased EBV EBNA1, LMP1/2, and EBER expression [103]. In Asian countries, CAEBV has a poor prognosis because of high T/NK cell involvement [104]. However, in Western countries, CAEBV has a relatively low mortality and disability rate because it mainly involves B cells in these patients [2]. Clinical attempts to treat CAEBV have not yet been unified, and treatments include traditional antiviral therapy, antitumor chemotherapy, and immunotherapy, with hematopoietic stem cell transplantation (HSCT) considered to be the only effective treatment [105].

### EBV-associated autoimmune diseases

EBV is also associated with the occurrence and development of various autoimmune-associated diseases, such as rheumatoid arthritis (RA), Sjögren’s syndrome (SS) and systemic lupus erythematosus (SLE). EBV infection can activate and modulate the immune system, thereby increasing the risk of autoimmune diseases. Defective EBV-specific T cells, increased viral load and expression of lytic phase proteins, and high levels of EBV antibodies in patients with RA, SS, and SLE all support an etiological role for EBV infection in the development of autoimmune diseases [106]. Recent research suggested that there are several mechanisms by which EBV causes autoimmune diseases. First, EBV can infect lymphocytes and express immune regulatory proteins that are involved in immune evasion, which can impact the host immune system [107–109]. Second, EBV can induce the production of many cytokines and inflammatory factors. Virus-encoded EBER can form complexes with the cellular EBER-binding protein La (SSB) and can release a large amount of proinflammatory factors by mediating the TLR3 signaling pathway, thereby enhancing the self-reactivity of nuclear ribonucleoprotein La in patients with SS and SLE [56]. Finally, EBV can cause the loss of immune tolerance and promote the progression of autoimmune diseases through molecular mimicry [110]. Most RA patients produce characteristic autoantibodies, including rheumatoid factor (RF) and anti-citrullinated protein antibodies (ACPAs). Studies have revealed that latent EBV transcripts and latent and lytic EBV proteins are detected in ectopic lymphoid structures resembling germinal centers in RA synovium [111], and antibodies against EBNA2 citrullinated peptides are detected in RA patients. Therefore, EBV can induce an immune response in the body, which can then be redirected toward self-antigens through cross-reactivity and epitope spreading [112].

### EBV-associated malignant tumors

#### Burkitt lymphoma

EBV is a human lymphoma virus that can cause many types of lymphomas, such as Burkitt lymphoma (BL) and Hodgkin lymphoma (HL). BL is the earliest lymphoma confirmed to be associated with EBV infection. BL mostly occurs in children, is aggressive and highly malignant, and progresses rapidly [113]. The WHO classifies BL into three categories: endemic, sporadic, and immunodeficiency-associated BL. Endemic BL (eBL) occurs mainly in children in equatorial Africa and has been shown to be associated with EBV infection [114]. eBL is characterized by EBV infection and translocation and dysregulation of the proto-oncogene *MYC*. Studies have shown that the dysregulated expression of activation-induced cytidine deaminase (AID) can lead to the translocation of *MYC* in cells infected with latent EBV, thereby promoting the occurrence and development of BL [115]. Moreover, the region is also a geographical area where *Plasmodium falciparum (P. falciparum*)-induced malaria is fully endemic. *P. falciparum* can not only cause dysregulated expression of AID but also can increase the number of B cells in the germinal center and increase the susceptibility of these cells to EBV infection [116]. In addition, EBV can also translocate *MYC* by inducing the expression of EBNA1, BHRF1, and EBER, with LMP1 inhibiting the proapoptotic protein Bcl-2-interacting mediator (BIM), thereby preventing the apoptosis of B cells [117]. The clinical manifestations of BL vary depending on the location of the disease in the body and can manifest as lymph node enlargement, maxillofacial mass, and acute abdomen pain caused by an abdominal mass. Bone marrow metastases can proceed rapidly, and these patients may present with leukemia-like symptoms. The main treatment option is chemotherapy, but the CHOP regimen is not effective. Combination treatment with rituximab can improve long-term survival, and complete remission may be achieved through allogeneic HSCT [118].

#### Hodgkin lymphoma

In 1987, Weiss et al. reported for the first time that the detection rate of EBV-DNA in HL tissues was 20-50% [119]. Immunohistochemistry and EBER in situ hybridization were then used to detect the presence of EBV in Hodgkin and Reed-Sternberg (HRS) tumor cells, confirming the link between EBV and HL [120]. The WHO classifies HL into two subtypes: classical HL (cHL) and nodular lymphocyte predominant HL (NLPHL), among which cHL is associated with EBV infection [121]. In EBV-positive cHL, EBV expression is usually restricted to the Latency II type, in which EBNA1, LMP1, LMP2A, and some noncoding RNAs are mainly expressed. LMP1 activates downstream NF-κB, JAK/STAT and PI3K signaling pathways by simulating CD40 receptors, thereby inducing in germinal center B cells transcriptional changes characteristic of HRS cells [122]. HRS cells can also downregulate B-cell-specific marker expression [123]. Approximately 25% of EBV-positive HL patients harbor deleterious mutations in the B-cell antigen receptor (BCR) that induce B-cell death [124], and LMP2A acts as an alternative BCR receptor in HRS cells, allowing survival of BCR-deficient B cells [125]. In 90% of the clinical cases of HL, lymph node enlargement is the first symptom, which gradually spreads from a single lymph node group to systemic lymph nodes. Late-stage HL is associated with liver, spleen, bone marrow and other organ involvement. A total of 20–30% of patients may experience unexplained fever, night sweats, weight loss, fatigue, itching and other symptoms. The treatment of cHL is usually based on a combination of radiotherapy and chemotherapy. After remission, autologous HSCT can be considered as consolidation therapy, and some relapsed or refractory patients can be considered for treatment with biologics.

#### Nasopharyngeal carcinoma

EBV is one of the main causes of nasopharyngeal carcinoma (NPC), especially in high-incidence areas in southern China and Southeast Asia. The WHO classifies NPC into three subtypes [126]: Type I (keratinizing squamous cell carcinoma), Type II (nonkeratinizing squamous cell carcinoma), and Type III (undifferentiated carcinoma), with progressively lower degrees of differentiation and increasing association with EBV infection [127]. Early detection of nasopharyngeal carcinoma is very difficult because onset is usually not apparent, and the malignancy rate is high, with 70% of patients in an advanced stage when they first seek medical attention [128]. EBV in the latent phase infects the malignant NPC epithelial cells following the Latency Type II pattern and expresses EBNA1, LMP1/2, EBER, and some miRNAs [129]. Studies have revealed that LMP1 can not only promote cell growth but also inhibit apoptosis [130, 131]. However, its expression also correlates with the characteristics of NPC metastasis. Studies have found that LMP1 can increase the invasiveness of tumor cells by affecting the expression of matrix metalloproteinase9 (MMP9) [132], mucin1 [133] and ezrin protein [134]. In addition, LMP1 can affect the degradation of the matrix around a tumor to promote the invasion and metastasis of NPC [135]. A study found that LMP1 promotes lymphangiogenesis and NPC lymph node metastasis by activating the vascular endothelial growth factor-C (VEGF-C)/VEGF receptor 3 axis [136]. In addition, BART miRNAs play important roles in the development of NPC, which may be related to their interference with apoptosis and evasion of T-cell-related immunity [137, 138]. Cervical lymph node metastasis is the most common clinical manifestation of NPC and may be accompanied by bloody saliva or nasal secretions, nasal congestion, ear discomfort, and headache [139]. To date, the treatment of NPC has been predominately radiotherapy and chemotherapy.

#### Gastric cancer

In 2014, The Cancer Genome Atlas (TCGA) proposed a new molecular classification of gastric cancer. Gastric cancer was first classified into four types at the molecular level: The EBV-infected type, genomically stable type, chromosomal instability type, and microsatellite unstable type [140]. Nearly 10% of gastric cancer cases worldwide are associated with EBV infection. EBV expression is usually restricted to the Latency Types I and II patterns, with EBNA1, LMP1, and LMP2A mainly expressed [141]. EBV-associated gastric cancer (EBVaGC) presents with molecular features including recurrent mutations in PIK3CA, DNA hypermethylation, and amplification of JAK2 and PD-L1/2 [140]. PIK3CA mutation activates the PI3K/AKT signaling pathway to promote tumor cell proliferation [142]; DNA hypermethylation silences many tumor suppressor genes [143]; overexpression of PD-L1 facilitates tumor cell immune escape and so on [144]. EBVaGC is more common in men and is the type associated with significant lymphocytic infiltration [145] and better prognosis [146]. At present, the treatment of EBVaGC is based on surgical resection supplemented with radiotherapy and chemotherapy.

## Conclusions and Perspectives

The infection rate of EBV in the population is extremely high. The relationship between the immune system and EBV is complex and dynamic. The immune system plays an important role in controlling viral replication, but EBV has evolved a series of mechanisms to evade immune detection and establish lifelong infection in a host. The pathogenic mechanism underlying EBV infection is complex and can affect various systems, exhibiting a variety of atypical clinical symptoms and signs and leading to various benign or malignant diseases. Effective treatments for EBV infection are currently lacking, and the most effective approach is the design of an EBV vaccine. Several vaccines based on targets involved in EBV invasion have shown promising experimental results. To date, there have been many studies on EBV glycoproteins used as antigens to develop prophylactic EBV vaccines, with most focusing on gp350. In 1995, China conducted the first clinical trial of a recombinant viral vector encoding gp350/220, and the results showed an increase in neutralizing antibody titers against EBV in vaccinated teenagers [147]. Glycoproteins gHgL, gp42, and gB have been identified as targets for neutralizing antibodies [148]. The approach to therapeutic EBV vaccines focuses on stimulating T cells to increase the immune control and clearance of EBV by the body. The first antigens targeted in therapeutic vaccines were EBNA1 and LMP2 proteins, which can significantly induce carcinogenesis. By inserting EBV antigen DNA sequences into the viral vector genome, specific CD8 + and CD4 + T-cell immune responses were increased [149]. The second approach involves using DCs harvested from a patient and pulsed with EBV peptides to induce functional CD8 + T-cell immunity, which significantly reduced the volume of EBV-positive NPC tumors [150]. The third approach involves the combination of the mentioned two for treatments. However, a mature anti-EBV vaccine is still not on the market. With the deepening of our understanding of the pathogenic mechanisms underlying EBV infection, it is believed that we will ultimately overcome the challenges caused by EBV infection in the future.

## Data Availability

The source data and materials presented in the review are available from the corresponding author upon reasonable request.

## Abbreviations

EBV: Epstein‒Barr virus
the WHO: the World Health Organization
EBNA: EBV nuclear antigen
LCLs: lymphoblastoid cell lines
IM: infectious mononucleosis
CAEBV: chronic active EBV infection
CR: complement receptor
IE: immediate early
E: early
L: late
CTLs: cytotoxic T lymphocytes
LMPs: latent membrane proteins
EBERs: EBV-encoded RNAs
miRNAs: microRNAs
ZTA: transcription factor Z
RTA: transcription factor R
PAMPs: pathogen-associated molecular patterns
DAMP: damage-associated molecular pattern
PRRs: pattern recognition receptors
TLRs: Toll-like receptors
RIG-I: retinoic acid-inducible gene-I
RLRs: retinoic acid-inducible gene-I (RIG-I)-like receptors
cGAMP: cyclic GMP-AMP
cGAS: cyclic GMP-AMP (cGAMP) synthase
IFI16: interferon gamma-inducible protein 16
IFN-1: type I interferon
MyD88: myeloid differentiation primary response 88
TRIF: TIR-domain-containing adapter protein-inducing interferon-β interleukin
TNF-α: tumor necrosis factor-α
IRF: interferon regulatory factor
dsDNA: double-stranded DNA
ATP: adenosine triphosphate
GTP: guanosine triphosphate
STING: stimulator of interferon genes
NK: natural killer
iNKT: invariant NKT
APC: antigen-presenting cell
DCs: dendritic cells
pDCs: plasmacytic dendritic cells
cDCs: classical myeloid dendritic cells
VCA: viral capsid antigen
IgM/A: immunoglobulin M/A
MHC: major histocompatibility complex
PTLD: posttransplant B lymphoproliferative disease
DUB: deubiquitinase
MAVS: mitochondrial antiviral signaling
TRIM: tripartite motif-containing protein
Tyk: tyrosine kinase
STAT: signal transducer and activator of transcription
ISG: interferon-stimulated gene
SAMHD: sterile alpha motif and HD domain
dNTPase: deoxynucleotide triphosphate hydrolase
PKR: dsRNA-dependent protein kinase
ULBP: UL16-binding protein
TAP: transporter associated with antigen processing
CIITA: Class II transactivator
HSCT: hematopoietic stem cell transplantation
RA: rheumatoid arthritis
SS: Sjögren’s syndrome
SLE: systemic lupus erythematosus
SSB: EBER-binding protein La
RF: rheumatoid factor
ACPA: anti-citrullinated protein antibody
BL: Burkitt lymphoma
HL: Hodgkin lymphoma
eBL: endemic BL
BIM: Bcl-2-interacting mediator
cHL: classical Hodgkin lymphoma
NLPHL: nodular lymphocyte predominant Hodgkin lymphoma
BCR: B-cell antigen receptor
NPC: nasopharyngeal carcinoma
MMP9: matrix metalloproteinase9
VEGF-C: vascular endothelial growth factor-C
TCGA: The Cancer Genome Atlas
EBVaGC: EBV-associated gastric cancer
